# Ontogenic Caste Differences in the Van der Vecht Organ of Primitively Eusocial Neotropical Paper Wasps

**DOI:** 10.1371/journal.pone.0154521

**Published:** 2016-05-11

**Authors:** André Rodrigues de Souza, Iacopo Petrocelli, José Lino-Neto, Eduardo Fernando Santos, Fernando Barbosa Noll, Stefano Turillazzi

**Affiliations:** 1 Departamento de Entomologia, Universidade Federal de Viçosa, 36570–000, Viçosa, Minas Gerais, Brazil; 2 Dipartimento di Biologia Evoluzionistica ‘Leo Pardi’, Università degli Studi di Firenze, Via Romana 17, 50125, Firenze, Italy; 3 Departamento de Biologia Geral, Universidade Federal de Viçosa, 36570–000, Viçosa, Minas Gerais, Brazil; 4 Departamento de Zoologia e Botânica, Instituto de Biociências, Letras e Ciências Exatas, Universidade Estadual Paulista “Júlio de Mesquita Filho”, 15054-000, São José do Rio Preto, São Paulo, Brazil; Charles University in Prague, CZECH REPUBLIC

## Abstract

Recent studies have reported incipient morphological caste dimorphism in the Van der Vecht organ size of some temperate *Polistes* paper wasps. Whether species other than the temperate ones show a similar pattern remains elusive. Here, we have studied some Neotropical *Polistes* species. By comparing females collected through the year, we showed caste related differences in the size of the Van der Vecht organ in *P*. *ferreri* (body size corrected Van der Vech organ size of queens = 0.45 ± 0.06, workers = 0.38 ± 0.07 mm^2^, *p* = 0.0021), *P*. *versicolor* (body size corrected Van der Vech organ size of queens = 0.54 ± 0.11, workers = 0.46 ± 0.09 mm^2^, *p* = 0.010), but not *P*. *simillimus* (body size corrected Van der Vech organ size of queens = 0.52 ± 0.05, workers = 0.49 ± 0.06 mm^2^, *p* = 0.238). Therefore, it seems that queens and workers of some Neotropical *Polistes* have diverged in their ontogenic trajectory of the Van der Vecht organ size, providing clear evidence for incipient morphological caste dimorphism. As *Polistes* are distributed mostly in the tropics, we propose that physical caste differences may be widespread in the genus. Also, we highlight that morphological divergence in the queen–worker phenotypes may have started through differential selection of body structures, like the Van der Vecht organ.

## Introduction

On observing a typical colony full of females of primitively eusocial Hymenoptera, such as ponerine ants, halictid bees, stenogastrine wasps and *Polistes* paper wasps, one can comprehend that all colony members are morphologically similar. However, if each individual receives a unique combination of paint on the thorax [1], it is possible for an observer to follow the behavior of each colony member. This helps identify that within the colony, one or a few individuals are the most aggressive ones, forage less, and lay most of the eggs, even as most of individuals are less aggressive, forage at higher rates and lay no or only a few eggs, like *Polistes* wasps [2, 3]. These two behavioral groups constitute examples of castes, broadly defined as subgroups of colony members, genetically, but not physiologically or anatomically homogeneous, that specialize in particular tasks for prolonged periods of time [4]. Although in colonies of primitively eusocial insects the queen–worker morphology greatly overlaps, meaning that there is a lack of morphological castes, in colonies of highly eusocial insects there is little or no overlap between the queen–worker morphology, so that physical castes do exists [5, 6].

In the temperate species of the primitively eusocial *Polistes* paper wasps, colonies are started in the spring by queens who emerged and mated in the summer of the previous year, and then subsequently overwintered. Colonies produce generations of workers followed by the future queens and males, all reared in annual nests made of wood pulp [7]. Tropical species follow a similar pattern [8, 9], but one striking difference is that only the temperate species necessarily have to overwinter. Generally, in the temperate species, only the queens survive overwintering, as this requires larger fat bodies, a common trait in queens [10], but not in the workers. The larger fat bodies in queens lead them to have a wider and heavier abdomen than workers [11]. Also, in wasps studied in both temperate and tropical regions, workers from the first generation are smaller than queens, but workers produced close to the end of the colony cycle have a size similar to the queens [7]. The queen–worker physical differences in *Polistes* have long been considered to be a consequence of isometric growth, that is, proportional relationships between body parts are the same in bigger and smaller individuals [8, 11, 12]. Keeping this in view, *Polistes* queens would be bigger versions of workers. However, this may not always be the case. Recent studies on the females of this genus suggest that changes in the wasp`s overall body size may lead to a disproportional increase in the dimensions of specific body structures, called allometric growth [13, 14]. The Van der Vecht organ is a hairy, slightly sclerotized cuticular area at the anterior edge of the last gastral sternite of the *Polistes* paper wasp females. It is composed of numerous tegumental glands of the third type [15] clustered in two lateral masses, opening onto the external cuticle of this area [16]. The Van der Vecht organ secretion is formed by a complex blend of hydrocarbons [17] applied on the nest when females rub the abdomen on the nest pedicel and nest surface. The hydrocarbons produced by the Van der Vecht organ are thought to have a dual function: (i) to repel ants when applied on the nest pedicel [18–20], especially in the pre-worker stage, when there are just one or a few queens to defend the nest; and (ii) to induce dominance recognition from the queen to the subordinate individuals when applied on the nest surface [17, 19–22], thus preventing the latter’s ovary development. Therefore, selection on the queen’s Van der Vecht organ may have been different from that of the workers. Indeed, at least in some temperate *Polistes* species like *P*. *gallicus* [13], *P*. *dominula* [14] and *P*. *nimphus* [14], the queens show a hypermetric allometry of the Van der Vecht organ. corresponding to an increased functionality [23, 24] compared to workers. It means that changes in a wasp`s overall body size lead to a disproportional increase in the dimensions of the Van der Vecht organ and this relation differs among queens and workers. As a result, the authors [13, 14] conclude that despite the strong overlap between the queen–worker morphology, these wasps have some degree of physical caste dimorphism, at least with respect to the Van der Vecht organ size.

Morphological caste differences in the Van der Vecht organ size have only been investigated in a few temperate species [13, 14]. As *Polistes* are distributed mostly in the tropics [25], we do not have, to date, a good knowledge on the occurrence of morphological caste differences within the genus in the Neotropical species. Given that, (i) Van der Vecht organ compounds are used to repel ants, specially at the pre-worker stage [18–20], (ii) the increased Van der Vecht organ area corresponds to an increased functionality [23, 24], and (iii) ant predation on paper wasp nests is higher in the tropics than in temperate areas [26–30], we predict that, just as in temperate *Polistes*, the Neotropical ones would also have a caste-dependent difference in the Van der Vecht organ size.

Here, we study three common widespread tropical paper wasps, *Polistes ferreri*, *Polistes simillimus*, and *Polistes versicolor*. Behavioral evidence suggests that these wasps may use the Van der Vecht organ compounds in a similar manner as their temperate congenerics, as they also rub the abdomen across both nest pedicel and nest surface, probably applying the Van der Vecht organ compounds [3, 31, 32]. Therefore, we aim to verify, whether the size of the Van der Vecht organ increases with the wasps`overall body size in these Neotropical species and whether this change differs among castes.

## Materials and Methods

### Ethical Statement

The collection of wasps complies with the current laws in Brazil. No specific permits are required for collection of wasps, and the species used in the experiments are not endangered or protected in Brazil.

### Study site and collections

*Polistes* colonies were located from March to December 2013 in the public gardens of Juiz de Fora, Minas Gerais State, southeastern Brazil (21°46`S, 43°21`W, 800 m asl). During this period, we collected both queens and workers. We sampled 16 workers and 18 queens from 22 colonies of *P*. *ferreri*, 15 workers and 26 queens from 29 colonies of *P*. *simillimus*, and 24 workers and 27 queens from 34 colonies of *P*. *versicolor*.

### Caste assignment

To assign wasps as queens or workers, we combined different approaches, based on the developmental stage of the colony each wasp came from [33], as well as the wasp`s wing wearing [8], fat bodies [7, 10, 34–37], and reproductive status [37–39]. Thus, queens collected early in the colony cycle (pre-worker colonies) had multistratified fat bodies, developed ovaries (many mature oocytes), and they were all inseminated. Queens collected late in the colony cycle (the period during which the colony produces reproductive forms, males, and future queens) had no evidence of wing wearing, which was expected of future queens who had not started nest-building activities. Also, these future queens had multistratified fat bodies, but ovary development or insemination was variable. Workers collected from worker producing colonies had some degree of wing wearing. Dissection of these individuals showed that they had unistratified fat bodies, undeveloped ovaries, and no insemination. Therefore, we were able to sample females from different generations, comprising a proper sample for our comparisons.

### Light microscopy of the Van der Vecht organ

To study light microscopy of the organ, we dissected female wasps’ last sternites and processed them as following. To observe the secretory cells, some sternites were fixed in ethanol-acetic acid (3:1) for 2 h and then submitted to Feulgen reaction (20 min hydrolyses in 5 N HCl at ambient temperature, 2 h in Schiff’s reagent). These sternites were placed on histological slides and covered with coverslips in a drop of 50% sucrose. To have a general sense of the organ histology, some sternites were fixed for 4–12 h in 2.5% glutaraldehyde solution in 0.1 M phosphate buffer, pH 7.2. Then, they were washed for 2 h in the same buffer, post-fixed in 1% osmium tetroxide for 2 h, dehydrated in different concentrations of ethanol and included in historesine (Leica Historesin). Semi-thin sections (2 μm thick) were placed on histological slides and stained with toluidine blue.

To collect morphometric data of the cuticular excretory area of the Van der Vecht organ, queen and worker sternites were diaphonized for one hour in Xylol. After this, they were flatted against histological slides and coverslips in a drop of 50% sucrose. We took a picture of each sternite. Also, we removed the head of each wasp and took a picture of it in frontal view.

All pictures were acquired with a Panasonic digital camera mounted on an Olympus BX-60 microscope.

### Measurements

**Fig 1:**
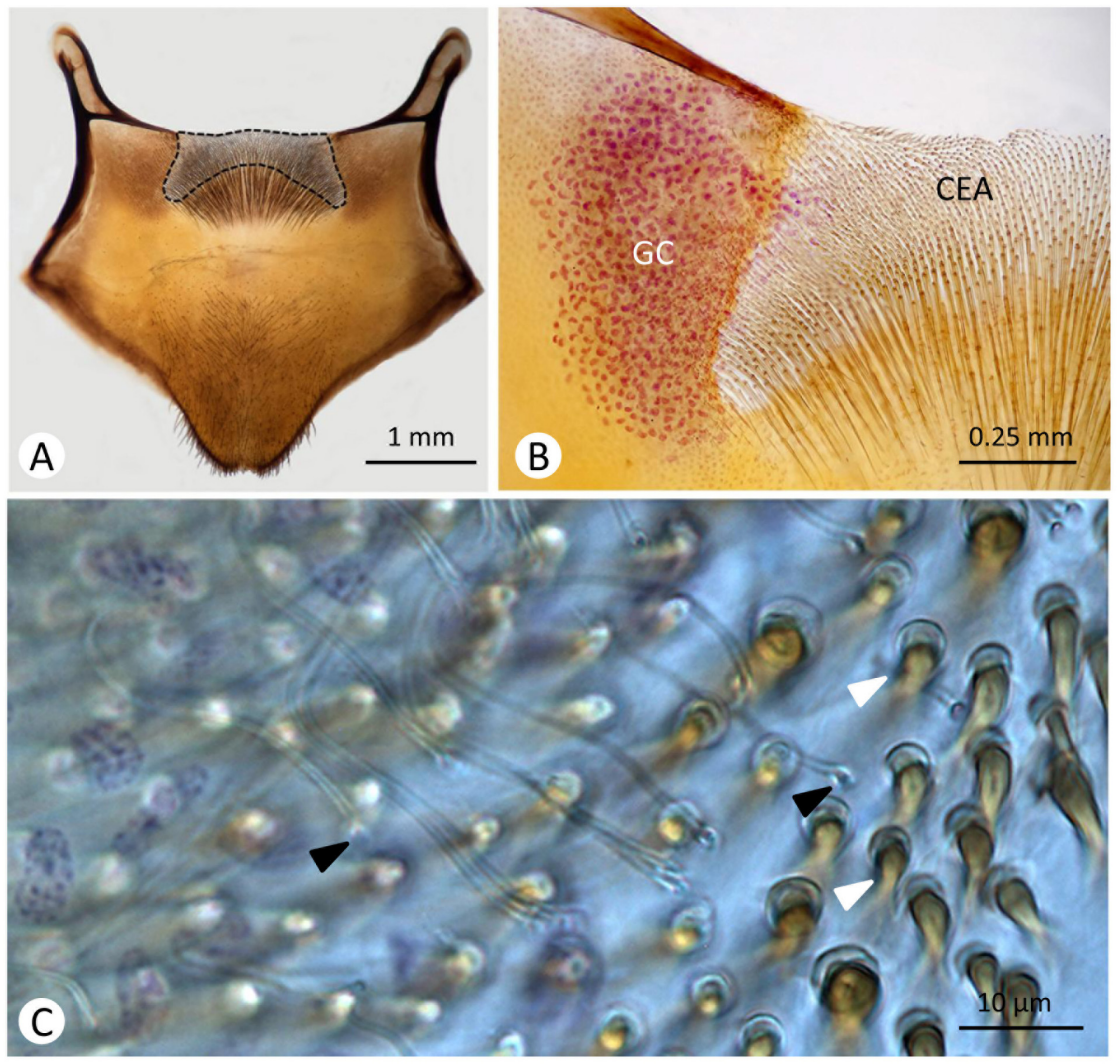
The last gastral sternite of *P*. *versicolor* female, showing the Van der Vecht organ. **A)** Internal frontal view of a xylol treated sample, showing the cuticular excretory area (dashed line) on the anterior medial edge of the sternite. **B)** Half section of the Van der Vecht organ stained with Feulgen, showing one of the two lateral masses of tegumental gland cells (GC). CEA = cuticular excretory area. **C)** Magnification of the cuticular excretory area, showing that this is the area where the openings of the glandular cell ducts occur (black arrow heads). The white arrow heads indicate the hairs associated with the cuticular excretory area.

**Fig 2:**
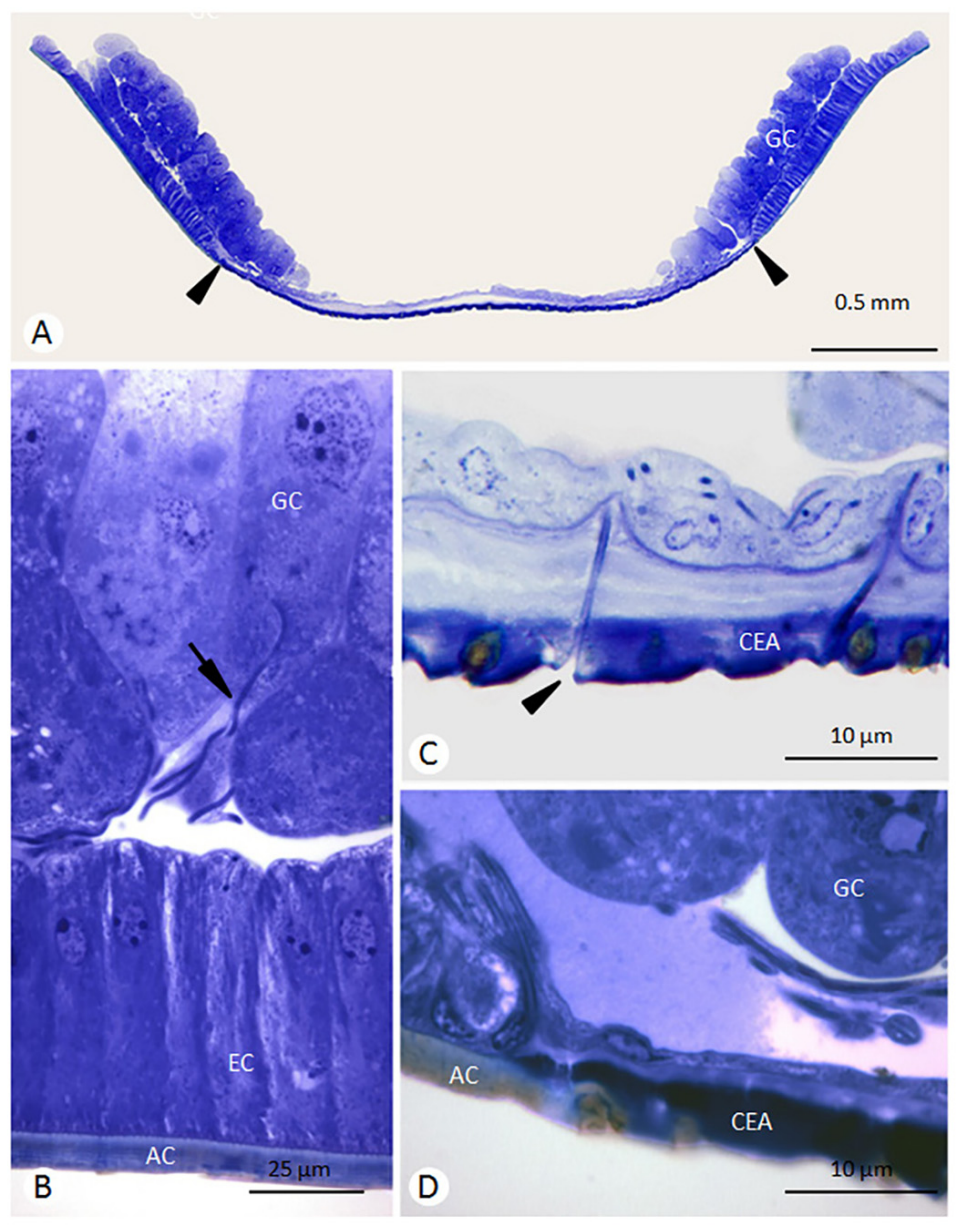
Histological sections of *P*. *versicolor* Van der Vecht organ stained with toluidine blue. **A)** Transversal section showing the two masses of tegumental glandular cells (GC) and the cuticular excretory area (between the black arrow heads). **B-D)** Magnifications showing a glandular cell with its excretory duct (black arrow in B), the funnell-shaped duct orifice (black arrow head in C) opening in the cuticular excretory area (CEA), and the transition region between the cuticular excretory area and the adjacent cuticle (AC). EC = Epithelial cells.

We measured the cuticular excretory area of the Van der Vecht organ (Fig 1a), this is, the area where the duct openings of the gland cells occur (Figs 1 and 2). Also, we measured the maximum head width, which is a good predictor of the wasp`s overall body size [38]. We used the IMAGE-J software (available at http://rsbweb.nih.gov/ij/). These data are fully available in S1 Table.

### Data analyses

We used *t* tests (preceded by *F* tests for variance) to compare the Van der Vecht organ size, as also the head width and the body size corrected Van der Vecht organ size (Van der Vecht organ size divided by the head width) in the queens and workers of each species. Then, we used Linear Models (LMs) to investigate whether, in each species, the Van der Vecht organ area was associated with the wasps’ overall body size (inferred from head width), caste (queen or worker), and their interaction. In each model, the Van der Vecht organ area was entered as a dependent variable, while head width, caste, and their interaction were entered as independent ones whenever they were used. In the variable “caste”, we treated numerically queens as 1 and workers as 2. Before running the models, we applied the quadratic transformation on the Van der Vecht organ area, to adjust its linearity to the independent variables. We chose the best-fit model by comparing multiple and simple linear models with the Akaike Information Criterion (AIC) [40] combined with the significance level of each model (see S2 Table). We checked outliers for each regressive model based on the Cook’s distance [41]. Specifically, we removed from the analyses, points with standardized residual and average values outside the intervals of 0.5 established by the Cook’s distance (see S2 Table). All statistical analyses were performed with the program R version 3.1.2 [42].

## Results

**Fig 3:**
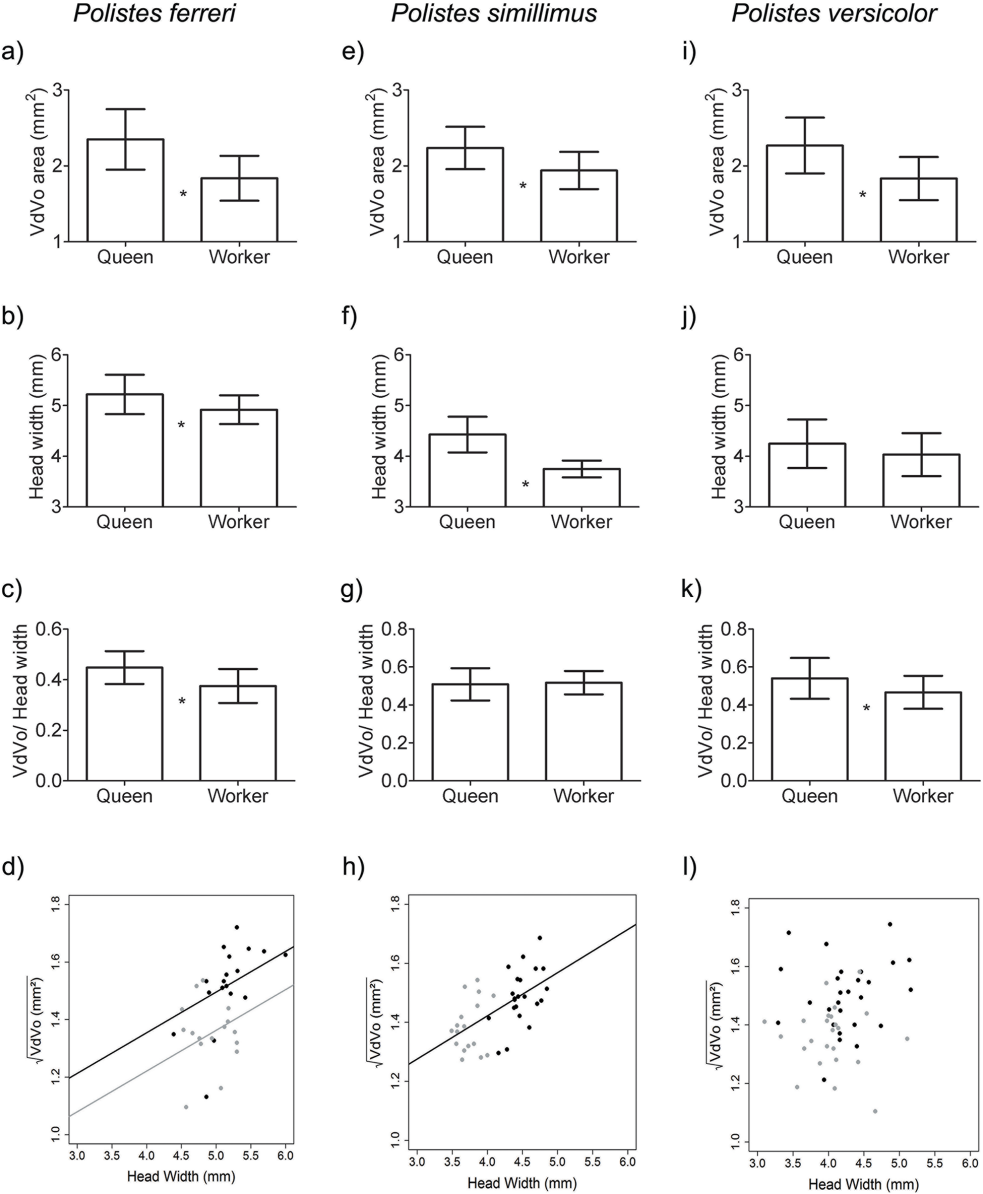
The relation between Van der Vecht organ size (VdVo), body size (inferred from head width), and castes in primitively eusocial Neotropical paper wasps. Data refer to the following species: *P*. *ferreri* (a–d), *P*. *simillimus* (e–h), and *P*. *versicolor* (i–l). In the column graphics we have presented the mean and standard deviation.* indicates statistical differences between castes (p<0.05). In the distribution graphics (d, h, l), each dot refers to a queen (black) or a worker (gray), while the lines represent the best-fit linear model (see S2 Table for more details).

In *P*. *ferreri*, queens have a bigger Van der Vecht organ area than the workers (Fig 3a, Table 1). Given that the queens also have a bigger overall body size, measured as head width (Fig 3b, Table 1), the Van der Vecht organ differences among castes could be explained if proportional relationships between body parts are the same in bigger and smaller individuals, leading to an isometric growth (if queens are bigger versions of workers). However, the queen–worker differences remain consistent even after correcting for wasp body size (Fig 3c, Table 1), suggesting that factors other than body size are involved. Indeed, the LM analyses suggest that in addition to body size, caste also plays a role in explaining the Van der Vecht organ size (Fig 3d, Table 2). It means that the effect of the overall body size on the Van der Vecht organ size is different in queens and workers, thus providing evidence for caste-dependent dimorphism in *P*. *ferreri* Van der Vecht organ size.

**Table 1:** Descriptive (Mean±SD) and inferential statistics of wasp body measurements.

		Caste		Variance test		t test		
		Queen	Worker	*F*	*P*	*t*	*df*	*p*
*P. ferreri*	Van der Vecht organ area (mm^2^)	2.35 ± 0.40	1.84 ± 0.30	1.819	0.247	4.250	33	0.0001
	Head width (mm)	5.22 ± 0.39	4.92 ± 0.28	1.870	0.226	2.578	33	0.0146
	Van der Vecht organ area/Head width	0.45 ± 0.06	0.38 ± 0.07	0.935	0.882	3.343	33	0.0021
*P. simillimus*	Van der Vecht organ area (mm^2^)	2.22 ± 0.28	1.94 ± 0.25	1.292	0.597	3.389	38	0.0017
	Head width (mm)	4.49 ± 0.21	3.75 ± 0.17	1.618	0.317	12.002	38	0.0001
	Van der Vecht organ area/Head width	0.52 ± 0.05	0.49 ± 0.06	0.769	0.561	-1.200	38	0.238
*P. versicolor*	Van der Vecht organ area (mm^2^)	2.27 ± 0.37	1.86 ± 0.29	1.575	0.275	4.370	49	0.0001
	Head width (mm)	4.25 ± 0.48	4.03 ± 0.42	1.271	0.565	1.702	49	0.095
	Van der Vecht organ area/Head width	0.54 ± 0.11	0.46 ± 0.09	1.532	0.305	2.673	49	0.010

**Table 2:** Statistics of the best fit linear model explaining the size variation in the Van der Vecht organ of three *Polistes* paper wasps. For additional details, check S2 Table.

	*F*	adj. R^2^	*P*
*P*. *ferreri*	*F*_*2*,*32*_ = 12.27	0.399	0.00011
*P*. *simillimus*	*F*_*1*,*38*_ = 20.76	0.336	< 0.0001
*P*. *versicolor*	*F*_*1*,*49*_ = 18.98	0.264	< 0.0001

Similar to *P*. *ferreri*, *P*. *simillimus* queens have a bigger Van der Vecht organ area (Fig 3e, Table 1) and bigger body size (Fig 3f, Table 1) than workers, so here too an isometric growth could explain caste differences in the Van der Vecht organ size. Indeed, after correcting for wasp body size, the queen–worker differences in the Van der Vecht organ size no longer exist (Fig 3g, Table 1), suggesting that the body size is the main factor explaining caste differences in the Van der Vecht organ size. This is further supported by the LM analyses indicating that the Van der Vecht organ size is associated only with wasp body size (Fig 3h, Table 2). Therefore, we found no evidence for caste-dependent dimorphism in *P*. *simillimus* Van der Vecht organ size.

In *P*. *versicolor*, the queens have a bigger Van der Vecht organ area than the workers (Fig 3i, Table 1), but both castes have a similar body size (Fig 3j, Table 1). It means that the body size under isometric growth is unlikely to explain caste differences in the Van der Vecht organ size. Not surprisingly, the queen–worker differences in the Van der Vecht organ size remain consistent even after correcting for wasp body size (Fig 3k, Table 1), suggesting that factors other than wasp body size are involved. Indeed, the LM analyses suggest that the Van der Vecht organ size is associated with caste (Fig 3l, Table 2). Therefore, we found evidence for caste-dependent dimorphism in *P*. *versicolor* Van der Vecht organ size.

## Discussion

### Evidence for ontogenetic caste differences in the Van der Vecht organ size of Neotropical paper wasps

We provide evidence for caste-dependent dimorphism in the Van der Vecht organ size of *P*. *ferreri* and *P*. *versicolor*. Once wasps emerge to the adult stage, a further morphological change to hardened (sclerotized) body parts (cuticular structures) is impossible (unless by damage and wear), therefore, it can be considered that any differences in cuticular morphology among adults are the result of developmental processes during the immature stages. As the Van der Vecht organ is a cuticular structure [16], its difference among queens and workers of *P*. *ferreri* and *P*. *versicolor* is a clear indicator that morphological caste differentiation arises during the immature stages (pre-imaginally). Therefore, it seems that queens and workers of some Neotropical *Polistes* have diverged in the morphological ontogenetic trajectory of the Van der Vecht organ. The occurrence of ontogenetic caste difference in the Van der Vecht organ size of *Polistes* wasps is not universal [14], as illustrated by the contrasting results we reported for *P*. *simillimus*, *P*. *ferreri* and *P*. *versicolor*. These findings motivate research about why ontogenetic caste difference in the Van der Vecht organ size occurs in some species, but not in others. To date, only a few *Polistes* species have been studied, showing that such difference is not restricted by the mode of colony foundation or phylogeny [14]. Therefore, further investigation including *Polistes* living in different social and ecological contexts is needed if we are to understand the variation in the occurrence of morphological castes in *Polistes*.

### Morphological castes in *Polistes* are incipient

Despite the differences in the Van der Vecht organ size of queens and workers of some *Polistes*, it is important to note that there is a considerable overlap between the two caste phenotypes related to the Van der Vecht organ [13, 14]. As a result, previous authors have claimed that morphological castes in *Polistes* wasps are incipient [13, 14]. This differs from that observed in highly eusocial species, as they have a strong and discrete morphological caste dimorphism [5, 6]. The incipient caste dimorphism may represent an intermediate step between the complete absence of morphological castes and the occurrence of discrete morphological castes in the eusocial species.

### The evolution of queen–worker differences in the Van der Vecht organ size

The Van der Vecht organ secretions in temperate *Polistes* are thought to repel ants when applied on the nest pedicel [18–20], especially in the pre-worker stage, when there are just one or a few queens to defend the nest; and to induce dominance recognition from the queen to the subordinate individuals, preventing the latter`s ovary development, when applied on the nest surface [17, 19, 20, 22]. Given these functions, the ontogenetic differences in the Van der Vecht organ size of temperate *Polistes* have been proposed to be an adaptative solution for queens to deal with nest defense at the beginning of the colony cycle and to deal with intraspecific communication. We suspect that this may also be the case for tropical *Polistes*, because (i) females of *P*. *ferreri*, *P*. *simillimus*, and *P*. *versicolor* also rub their abdomen across both the nest pedicel and nest surface, probably applying the Van der Vecht organ compounds [3, 31, 32] and because (ii) in tropical environments, ant predation on paper wasp nests is high [26–30]. Therefore, one likely possibility is that the selection may have favored an increase in some Neotropical *Polistes* queens’ Van der Vecht organ in response to high levels of predation by ants.

### Caste differences in the Van der Vecht organ size of other primitively eusocial wasps

Previous studies suggested that morphological caste dimorphism in *Polistes* Van der Vecht organ is a flexible trait, because it is not constrained by the wasps’ nest foundation strategies or phylogeny [14]. We add to this idea by showing that morphological castes in this taxon are not restricted to temperate species. Given that *Polistes* is distributed mostly in the tropics [25], it seems that morphological caste difference in the wasps’ Van der Vecht organ is a general rather than an exceptional pattern in this genus. We highlight the possibility that caste morphological dimorphism may also occur in Neotropical wasps other than *Polistes*. For example, colonies of the primitively eusocial wasps *Mischocyttarus* start in a manner very similar to *Polistes* colonies. There is evidence that females of *Mischocyttarus drewseni* rub their abdomen across the nest surface and that their the Van der Vecht organ secretions repel ants [43], but it remains to be investigated whether or not there are morphological caste differences in the Van der Vecht organ size in this species.

### Implications for the study of morphological caste differences

Studies on species with moderate caste dimorphism, such as Epiponini wasps [44], have frequently used a number of external body measurements to access the degree of queen–worker morphological dimorphism. Increasing the number of measurements on body structures can improve the chance to find morphological differences among castes, but focusing on structures with a known function, such as the Van der Vecht organ, can also be useful. For example, a study by Gobbi et al. [8] compared the size of 17 body structures among queens and workers of *P*. *versicolor*, but they did not find morphological caste differences. For the same species, however, we found morphological caste differences in the Van der Vecht organ size. Thus, focusing on structures with a known function may be a promising avenue to study the morphological caste in paper wasps.

### *Polistes* wasps on the threshold of the evolution of morphological castes

The study of caste differences in social insects is recently receiving increased attention from a developmental perspective [45–53]. By showing ontogenetic morphological differences in body structures of queens and workers, we further add to the growing body of evidence suggesting that despite being primitively eusocial, *Polistes* wasps present a degree of ontogenetic (pre-imaginal) caste determination. For example, in addition to caste differences in fat bodies [7, 10, 34–37], studies have shown that differential gene expression prolonged larval development and the presence of storage hexamerine proteins clearly distinguishes the queens from the workers [35, 36, 44, 54, 55]. Also, *Polistes dominula* females have facial color patterns that work as visual signals associated with their individual quality, such as fighting ability [56]. These cuticular color signals are determined before adult emergence [57], and they are shown to be considerably different among queens and workers [58], suggesting the occurrence of some pre-imaginal caste bias in this species. Therefore, as highlighted by [14], the genus *Polistes* is on the threshold of the evolution of morphological castes. In this sense, we propose that at the early stages of eusociality, caste morphological dimorphism may have started by differential selection on queen and worker body structures, such as the Van der Vecht organ.

## Supporting Information

S1 Table Data used for statistical analyses. (XLSX)

S2 Table Parameters of models integrating the size variation in the Van der Vecht`s organ, caste and head width of three Neotropical species of *Polistes* paper wasps. (PDF)
