# Supplementary material for: Ontogenic Caste Differences in the Van der Vecht Organ of Primitively Eusocial Neotropical Paper Wasps
Source: PLoS One. 2016 May 11;11(5):e0154521. doi: 10.1371/journal.pone.0154521 (PMC4864236; doi:10.1371/journal.pone.0154521)
Supplement: S2 Table — (PDF) [file pone.0154521.s002.pdf]

<u>Electronic Supplementary Material</u>

Ontogenic caste differences in the Van der Vecht's organ of primitively eusocial Neotropical paper wasps

André R. De Souza[1,*], Iacopo Petrocelli[2], José Lino-Neto[3] , Eduardo Fernando santos[4], Fernando Barbosa Noll[4], Stefano Turillazzi[2]

[1]Departamento de Entomologia, Universidade Federal de Viçosa, 36570-000 Viçosa, Minas Gerais, Brazil.

[2] Dipartimento di Biologia Evoluzionistica ‘Leo Pardi’, Università degli Studi di Firenze, Via Romana 17, 50125, Firenze, Italy.

[3]Departamento de Biologia Geral, Universidade Federal de Viçosa, 36570-000 Viçosa, Minas Gerais, Brazil.

[4] Departamento de Zoologia e Botânica, Instituto de Biociências, Letras e Ciências Exatas, Universidade Estadual Paulista “Júlio de Mesquita Filho”, 15054-000, São José do Rio Preto, São Paulo, Brazil.

[*]Corresponding author: e -mail: andrebioufjf@gmail.com; phone: +55 31 991004208

ESM_1-Parameters of models integrating the size variation in the Van der Vecht`s organ (VdVo), caste (each queen was entered in the models as value 1 while each worker as value 2) and head width of three Neotropical species of *Polistes* paper wasps. DV = dependent variable; IVs = independent variables; *RSE* = residual standard error; *DF* = degree of freedom; *P* = probability of the regressive model; *Pr(>|t|)* = t-distribution probability of each estimated coefficient; AIC = Akaike information criterion; sqrt = square root; † = best-fit model.

<table>
<tr><th rowspan="2"></th><th rowspan="2">DV ~ IVs</th><th rowspan="2">RSE</th><th rowspan="2">DF</th><th rowspan="2">P</th><th colspan="4">Coefficients</th><th rowspan="2">AIC</th></tr>
<tr><th>Estimate</th><th>Std Error</th><th>t</th><th>Pr(>|t|)</th></tr>
<tr><td rowspan="4">P. ferreri</td><td>sqrt(VdVo) ~ Head*Caste</td><td>0.1147</td><td>31</td><td>< 0.0001*</td><td>Int.: -0.5947
Head: 0.4411
Caste: 1.0155
Head:Caste: -0.2292</td><td>Int.: 0.8907
Head: 0.1738
Caste: 0.6292
Head: Caste: 0.1253</td><td>Int.: -0.668
Head: 2.538
Caste: 1.614
Head:Caste: -1.830</td><td>Int.: 0.5093
Caste: 0.0164*
Head: 0.1167
Head:Caste: 0.0769</td><td>-46.504</td></tr>
<tr><td>sqrt(VdVo) ~ Head+Caste</td><td>0.1188</td><td>32</td><td>0.0001*</td><td>Int.: 0.9227
Head: 0.1412
Caste: -0.1331</td><td>Int.: 0.3367
Head: 0.0599
Caste: 0.0442</td><td>Int.: 2.740
Head: 2.356
Caste: -3.012</td><td>Int.: 0.01*
Head: 0.025*
Caste: 0.005*</td><td>-44.915†</td></tr>
<tr><td>sqrt(VdVo) ~ Head</td><td>0.1326</td><td>33</td><td>0.0013*</td><td>Int.: 0.3533
Head: 0.2151</td><td>Int.: 0.3108
Head: 0.0610</td><td>Int.: 1.137
Head: 3.526</td><td>Int.: 0.2639
Head: 0.0013*</td><td>-38.1804</td></tr>
<tr><td>sqrt(VdVo) ~ Caste</td><td>0.1268</td><td>33</td><td>0.0003*</td><td>Int.: 1.7024
Caste: -0.1757</td><td>Int.: 0.0662
Caste: 0.0430</td><td>Int.: 25.703
Caste: -4.086</td><td>Int.: < 0.0001*
Caste: 0.0003*</td><td>-41.3158</td></tr>
<tr><td rowspan="4">P. simillimus</td><td>sqrt(VdVo) ~ Head*Caste</td><td>0.0849</td><td>36</td><td>0.0008*</td><td>Int.: 0.0851
Head: 0.3127
Caste: 0.4006
Head:Caste: -0.0892</td><td>Int.: 0.9102
Head: 0.2137
Caste: 0.6065
Head:Caste: 0.1511</td><td>Int.: 0.093
Head: 1.463
Caste: 0.661
Head:Caste: -0.590</td><td>Int.: 0.926
Head: 0.152
Caste: 0.513
Caste:Head: 0.559</td><td>-78.0107</td></tr>
<tr><td>sqrt(VdVo) ~ Head+Caste</td><td>0.0841</td><td>37</td><td>0.0002*</td><td>Int.: 0.5747
Head: 0.1938
Caste: 0.0444</td><td>Int.: 0.3711
Head: 0.7060
Caste: 0.0585</td><td>Int.: 1.549
Head: 2.744
Caste: 0.758</td><td>Int.: 0.1299
Head: 0.0093*
Caste: 0.4532</td><td>-79.6256</td></tr>
<tr><td>sqrt(VdVo) ~ Head</td><td>0.0837</td><td>38</td><td>< 0.0001*</td><td>Int.: 0.8369
Head: 0.1461</td><td>Int.: 0.1339
Head: 0.0321</td><td>Int.: 6.250
Head: 4.556</td><td>Int.: < 0.0001*
Head: < 0.0001*</td><td>-81.0089†</td></tr>
<tr><td>sqrt(VdVo) ~ Caste</td><td>0.0911</td><td>38</td><td>0.0016*</td><td>Int.: 1.5869
Caste: -0.0985</td><td>Int.: 0.0444
Caste:0.0290</td><td>Int.: 35.759
Caste: -3.403</td><td>Int.: < 0.0001*
Caste: 0.0016*</td><td>-74.2144</td></tr>
<tr><td rowspan="4">P. versicolor</td><td>sqrt(VdVo) ~ Head*Caste</td><td>0.118</td><td>47</td><td>0.001*</td><td>Int.: 1.2651
Head: 0.0897
Caste: 0.0689
Head:Caste: -0.0503</td><td>Int.: 0.4758
Head: 0.1129
Caste: 0.3131
Head:Caste: 0.0756</td><td>Int.: 2.659
Head: 0.794
Caste: 0.220
Head:Caste: -0.666</td><td>Int.: 0.011*
Head: 0.431
Caste: 0.827
Head:Caste: 0.509</td><td>-67.3847</td></tr>
<tr><td>sqrt(VdVo) ~ Head+Caste</td><td>0.1173</td><td>48</td><td>0.0003*</td><td>Int.: 1.5600
Head: 0.0187
Caste: -0.1383</td><td>Int.: 0.1727
Head: 0.0370
Caste: 0.0339</td><td>Int.: 9.033
Head: 0.506
Caste: -4.082</td><td>Int.: < 0.0001*
Head: 0.6149
Caste: 0.0002*</td><td>-68.9061</td></tr>
<tr><td>sqrt(VdVo) ~ Head</td><td>0.1348</td><td>49</td><td>0.1938</td><td>Int.: 1.2090
Head: 0.0543</td><td>Int.: 0.1720
Head: 0.0413</td><td>Int.: 7.028
Head: 1.317</td><td>Int.: < 0.0001*
Head: 0.194</td><td>-55.7120</td></tr>
<tr><td>sqrt(VdVo) ~ Caste</td><td>0.1165</td><td>49</td><td>< 0.0001*</td><td>Int.: 1.6436
Caste: -0.1423</td><td>Int.: 0.0507
Caste:0.0327</td><td>Int.: 32.394
Caste: -4.357</td><td>Int.: < 0.0001*
Caste: < 0.0001*</td><td>-70.6343†</td></tr>
</table>
